# The International Prevalence Study on Physical Activity: results from 20 countries

**DOI:** 10.1186/1479-5868-6-21

**Published:** 2009-03-31

**Authors:** Adrian Bauman, Fiona Bull, Tien Chey, Cora L Craig, Barbara E Ainsworth, James F Sallis, Heather R Bowles, Maria Hagstromer, Michael Sjostrom, Michael Pratt, The IPS Group

**Affiliations:** 1 Centre for Physical Activity and Health, School of Public Health, University of Sydney, Sydney, Australia; 2 School of Sport and Exercise Sciences, Loughborough University, Loughborough, UK; 3 School of Population Health, The University of Western Australia, Australia; 4 Canadian Fitness and Lifestyle Research Institute, Ottawa, Canada; 5 Active Living Research, San Diego State University, San Diego, CA, USA; 6 Department of Exercise and Wellness, Arizona State University, Mesa, AZ, USA; 7 Department of Biosciences and Nutrition at Novum, Karolinska Institute, Stockholm, Sweden; 8 US Centers for Disease Control, (Physical Activity and Nutrition Branch), Atlanta, GA, USA; 9 IPS Collaborating research groups in each country (see Appendix 1)

## Abstract

**Background:**

Physical activity (PA) is one of the most important factors for improving population health, but no standardised systems exist for international surveillance. The International Physical Activity Questionnaire (IPAQ) was developed for international surveillance. The purpose of this study was a comparative international study of population physical activity prevalence across 20 countries.

**Methods:**

Between 2002–2004, a standardised protocol using IPAQ was used to assess PA participation in 20 countries [total N = 52,746, aged 18–65 years]. The median survey response rate was 61%. Physical activity levels were categorised as "low", "moderate" and "high". Age-adjusted prevalence estimates are presented by sex.

**Results:**

The prevalence of "high PA" varied from 21–63%; in eight countries high PA was reported for over half of the adult population. The prevalence of "low PA" varied from 9% to 43%. Males more frequently reported high PA than females in 17 of 20 countries. The prevalence of low PA ranged from 7–41% among males, and 6–49% among females. Gender differences were noted, especially for younger adults, with males more active than females in most countries. Markedly lower physical activity prevalence (10% difference) with increasing age was noted in 11 of 19 countries for males, but only in three countries for women. The ways populations accumulated PA differed, with some reporting mostly vigorous intensity activities and others mostly walking.

**Conclusion:**

This study demonstrated the feasibility of international PA surveillance, and showed that IPAQ is an acceptable surveillance instrument, at least within countries. If assessment methods are used consistently over time, trend data will inform countries about the success of their efforts to promote physical activity.

## Introduction

Physical inactivity is an established risk factor for cardiovascular disease, cancer and diabetes, which along with chronic respiratory disease account for more than 60% of all deaths [1]. More than 80% of chronic disease deaths occur within low and middle income countries [LMC] [2]. The most recent estimates suggest that almost 2 million deaths per year worldwide are attributable to inactivit[1], leading to physical activity being described as 'the best buy in public health.' [3]. Despite global concerns about non-communicable disease in LMC [4], increasing obesity, and rapid changes in patterns of work, transport and recreation, physical activity surveillance and monitoring is only carried out in a few countries [5].

There is a significant gap in international physical activity surveillance, compared to surveillance of other chronic disease risk factors [6]. This gap makes it difficult to estimate the impact of physical inactivity on health outcomes. The World Health Organization pooled prevalence estimates to estimate the attributable risk of physical inactivity in the global burden of non-communicable diseases [1,7]. The lack of comparable data, along with the recent Global Strategy for Diet, Physical Activity and Health [DPAS] [8], have created a compelling need for internationally-comparable measures of physical activity that can be used to quantify population levels of exposure and monitor trends over time within and among countries.

Although some countries conduct national physical activity surveillance, use of different questionnaires makes it difficult to assess inter-country differences in physical activity. In 1997, the International Physical Activity Questionnaire (IPAQ) was developed as a surveillance instrument to measure multiple domains of physical activity. This was the first effort to develop an instrument suitable for global physical activity surveillance. The goal was to identify a common questionnaire that all countries could use that would permit comparability among countries on various domains of physical activity. The IPAQ items capture moderate and vigorous intensity leisure-time, domestic, occupational and transport-related domains, which summate to total physical activity [9-11]. Assessing multiple domains of activity is particularly important in developing and transitional countries, where measures confined to leisure-time activity may miss substantial daily physical activity undertaken for the purpose of work or travel [5]. Sitting time is assessed separately by hours of sitting time per weekday and weekend day. Inactivity measures are useful for monitoring the effect of health promotion strategies that encourage people to sit less and engage more in ambulatory activity.

The aims of the International Prevalence Study on Physical Activity (IPS) were to collect and compare for the first time nationally representative prevalence estimates on physical activity from a diverse set of countries and to qualitatively assess the use of IPAQ in large-scale representative population physical activity data collection.

## Methods

Development and international validation of the IPAQ has been previously reported [11]. In brief, surveillance-oriented questionnaires were developed for adults aged 18 to 65 years. Reliability and validity studies were conducted in 12 countries across 6 continents using standardised methods, and demonstrated reasonable test-retest reliability (intra-class correlations range 0.7–0.8) and inter-method validity (median rho = 0.67), with criterion validity around rho = 0.3 based on comparisons with accelerometer data. Measurement properties were similar to those of other physical activity surveys used in developed countries [12] with the IPAQ offering broader applicability to a wide range of countries and cultures. The IPAQ short form instrument was considered brief enough for physical activity surveillance, flexible enough to be used in telephone interview or self-administered applications, and adaptable enough to apply across cultures [11]. IPAQ has been used for physical activity surveillance activities in individual countries and in the European Union [13,14].

The International Prevalence Study (IPS) was coordinated by an international group of scientists. The study involved 3 primary phases: [i] country recruitment and development of comparable study methods; [ii] within-country data collection with IPAQ nested within existing surveys or as a stand-alone survey and [iii] analysis of the pooled data using standardised protocols. A Data Management Centre (DMC) provided technical support to participating countries, collated country-specific data sets, and undertook pooled analyses. A statement of ethics approval was obtained from all centres. Informed and voluntary consent was provided verbally or in writing from all participants.

Countries were invited through existing international and regional global health and physical activity networks, key organizations (World Health Organisation; U.S. Centers for Disease Control and Prevention) and various non-communicable disease networks. Expressions of interest were received from 24 countries or large within-country regions during 2002–2004. The inclusion criteria required a representative population sample of at least 1500 adults, using comparable data collection methods in spring or autumn, and using approved cultural translations of the IPAQ instrument [see Appendix 2]. A manual of operations specified the required study protocols and indicated where modifications could be made to accommodate the local context.

Twenty countries or large within-country regions were approved for IPS, completed data collection and provided population-level data to the DMC. Details of the participating countries, mode of questionnaire administration, response rate, data collection period and sample size are provided in Table 1. Face-to-face IPAQ interviews were conducted in 6 countries, self-administered questionnaires in 8 countries and telephone interviews in 6 countries. The median country-level response rate was 61%, ranging from 28% in Belgium, (due to nesting IPAQ in a population survey with detailed anthropometric measures) to above 80%. The total sample sizes across the 20 countries ranged from 1,010 (India) to 11,449 (Czech Republic). Only data from adults aged 18–65 years were included, the age range for which IPAQ was designed and evaluated.

**Table 1:** Survey sampling procedures and response details from 20 countries; International Prevalence Study, 2002–2004.

**Country**	**Sampling Procedures**	**IPAQ Short Form Administration**	**Month Year**	**Response Rate**	**Sample Size 18–65 yrs**	**% male**	**% education> 13 years**
Argentina	A representative sample of Buenos Aires – Multistage stratified random selection of houses/apartments and blocks	Self	Jun 2003	72%	1203	44.9	46.6

Australia	A nationally representative sample – RDD with simple random sample of households	Telephone	Apr 2003	55%	2691	44.0	48.8

Belgium	A representative sample of Flanders – Random selection of municipalities/cities and inhabitants within municipality/city	Self	Mar–May, Sept–Oct 2003	28%	1969	51.9	45.1

Brazil	State of Sao Paulo representative sample – Simple random sample proportional to size	Interviewer	Mar–May 2003	85%	991	48.5	N/A

Canada	A nationally representative sample – RDD sample proportional to number of households in each province	Telephone	Sept–Nov 02 – Mar–May 03	51%	2669	45.3	62.3

China (Shanghai)	A representative sample of Shanghai – Multistage sampling of 3 communities, 5 neighborhoods within communities, and adults within households	Interviewer	Nov–Dec 2002	84%	1593	51.5	32.5

Colombia	A representative sample of Bogota DC – Multistage unequal probability selection proportional to size	Interviewer	Mar–May 2003	84%	3000	40.4	19.1

Czech Republic	9 academic worksites in 10 regions; a nationally representative sample – Simple random sample proportional to size. Students distributed questionnaires to permanent/temporary residences, partly randomly selected	Self	Nov 2002 May 2003	58%	7513	48.2	42.2

Hong Kong SAR, China	A nationally representative sample – Stratified by district	Interviewer	Oct–Dec 02 – Jan–Feb 03	48%	4886	48.9	14.1

India	Convenience sample of employees and their families from 2 worksite populations in Ghaziabad and Nagpur	Interviewer	Jan–Dec 2003	88%	1005	48.7	38.2

Japan	22 universities and 6 worksites from different regions of Japan, representing nearly all areas	Self	July 2003	90%	4959	38.4	29.2

Lithuania	A systematic random sample from 10 rural districts and the 5 largest Lithuanian cities – Respondents sampled at fixed intervals after random selection of a starting point	Interviewer	Apr–May 2003	77%	2227	41.4	58.0

New Zealand	A nationally representative sample – Simple random sample proportional to size	Telephone	Mar–Apr 2003	42%	1495	40.8	36.9

Norway	A nationally representative sample – Simple random sampling	Self	Oct 2003	41.3%	1645	47.3	45.8

Portugal	A nationally representative sample – Simple random sample proportional to size	Self	Apr–May 2002	>80%	1525	47.3	3.1

Saudi Arabia	A representative sample of Riyadh City – Simple random sample of telephone-equipped households	Telephone	Mar–May 2003	66%	988	65.4	38.3

Spain	A representative sample of Catalonia – Simple random sample proportional to size	Self	Oct–Nov 2002	62.4%	1580	44.9	43.5

Sweden	A nationally representative sample – Simple random sampling	Self	Oct–Dec 2002	59%	1290	45.9	30.8

Taiwan	A nationally representative sample – RDD sample proportional to number of households, in 7 stratified areas	Telephone	Sept–Nov 2004	48.3%	4846	47.6	40.8

USA	A nationally representative sample – Simple random sample proportional to size	Telephone	Sept–Nov 2002	30.9%	4671	42.8	61.4

Note: RDD = Random Digit Dial; CATI = computer-assisted telephone interview

IPAQ was designed to measure physical activity across all domains of leisure-time, work, transportation, and household tasks. The IPAQ short form asks respondents to report frequency and duration of walking, moderate-intensity and vigorous-intensity activity performed for at least 10 minutes duration per session. IPAQ also collects information on total sitting time, but these data are not reported here. Weekly minutes of walking, moderate-intensity and vigorous-intensity activity were calculated separately by multiplying the number of days/week by the duration on an average day. Reported minutes per week in each category were weighted by a metabolic equivalent (MET; multiples of resting energy expenditure) resulting in a physical activity estimate independent of body weight, expressed in MET-minutes/week and computed by multiplying METs by minutes/week [11]. The summary indicator was used to categorise a population into three levels of physical activity: "low" [physically inactive], "moderate" and "high" levels of physical activity. The "moderate" level nominally indicated meeting physical activity guidelines of 30 minutes of moderate intensity activity 5 days a week, 20 minutes of vigorous activity 3 days a week, or a combination [15]. Because guidelines were based mainly on leisure time activity but IPAQ assessed four domains, the "high" activity level was developed and reflects approximately twice the MET-minutes of the "moderate" level. These categories were based on standard scoring criteria .

• *Low*: Meets neither 'moderate' nor 'high' criteria.

• *Moderate*: Meets any of the following three criteria: (a) 3 days of vigorous activity of at least 20 minutes/day; (b) 5 days of moderate-intensity activity or walking of > 30 minutes/day for > 10 minutes at a time; or (c) 5 days of any combination of walking, moderate-intensity or vigorous-intensity activities achieving at least 600 MET-minutes/week.

• *High*: Meets either of two criteria: (a) vigorous-intensity activity on > 3 days/week and accumulating at least 1500 MET-minutes/week; or (b) >5 days of any combination of walking, moderate-intensity, or vigorous-intensity activities achieving at least 3000 MET-minutes/week.

For country specific prevalence estimates, data were weighted to the national population based on study design. For international comparisons, all prevalence rates were age standardised to the world population distribution to enable comparability for totals across countries with varying population distributions by age [16,17]. Data analyses were conducted using SAS software V8.02 [18].

## Results

Data were from 20 countries with a total of 52,746 individuals aged 18–65 years. For most countries there was a fairly even balance of males and females with the exception of Colombia, Japan and Lithuania (where around 60% of the samples were female), and Saudi Arabia (males 65%). Data on education level were available for 19 countries and showed a wide variation among countries (Table 1).

Table 2 shows the prevalence of physical activity levels in total, and Table 3 shows these data by gender for each country. High physical activity was most prevalent in New Zealand, the Czech Republic, the USA, Canada and Australia. Four countries, Belgium, Brazil, Japan and Taiwan reported less than a third of their populations in the high PA category. For most countries, males were more active, with only Argentina, Portugal and Saudi Arabia showing women more active than men. It was noted that more than half of the males in 12 countries and females in 14 countries did not achieve the high physical activity threshold. The prevalence of low physical activity ranged from 7% to 43% among males and from 6% to 49% among women.

**Table 2:** Prevalence* of International Physical Activity Questionnaire categories among 18–65 year-olds by country^†^; International Prevalence Study, 2002–2004

Country	Weighted Valid n ##	Total sample (%)		
		
		Low active	Moderate Activity	High active
Argentina	1189	26.7 (24–29)	35.2 (32–38)	38.0 (35–41)
Australia	2642	17.2 (16–19)	24.3 (23–26)	58.6 (57–60)
Belgium	1922	43.0 (41–45)	27.4 (25–29)	29.6 (28–32)
Brazil	981	30.4 (28–33)	45.0 (42–48)	24.6 (22–27)

Canada	2626	13.7 (12–15)	26.7 (25–28)	59.6 (58–62)
China	1593	6.9 (6–8)	35.4 (33–38)	57.7 (55–60)
Colombia	2974	19.8 (18–21)	27.5 (26–29)	52.7 (51–55)
Czech Rep	7468	9.9 (9–11)	27.2 (26–28)	62.9 (62–64)

Hong Kong	4657	15.3 (14–16)	50.6 (49–52)	34.1 (33–35)
India	1004	23.4 (21–26)	38.7 (36–42)	37.9 (35–41)
Japan	4618	43.3 (42–45)	35.4 (34–37)	21.2 (20–22)
Lithuania	2210	15.0 (14–16)	32.9 (31–35)	52.1 (50–54)

New Zealand	1449	12.2 (10–14)	24.7 (22–27)	63.1 (61–66)
Norway	1625	26.1 (24–28)	33.6 (31–36)	40.3 (38–43)
Portugal	1435	26.2 (24–28)	28.5 (26–31)	45.3 (43–48)
Saudi Arabia	974	40.0 (37–43)	33.8 (31–37)	26.2 (23–29)

Spain	1541	24.2 (22–26)	36.2 (34–39)	39.6 (37–42)
Sweden	1254	23.9 (22–26)	37.3 (35–40)	38.8 (36–41)
Taiwan	4773	42.3 (41–44)	32.9 (32–34)	24.8 (24–26)
USA	4587	15.9 (15–17)	22.1 (21–23)	62.0 (61–63)

* Age and gender standardised to the world population 2002 [US Census Bureau 2002; Doll R et al 1966]

† Australia: Age 18–64 only; Hong Kong: Age 20–64 only; Japan: Age 18–39 only; Portugal: Age 40–65 only

## note that sample sizes differ from Table 1 due to minimal missing data on some physical activity questions

**Table 3:** Gender specific Prevalence* of International Physical Activity Questionnaire categories among 18–65 year-olds by country^†^; International Prevalence Study, 2002–2004

Country	Weighted Valid n ##	Men (%)			Women (%)		
		
		Low active	Moderate Activity	High active	Low active	Moderate activity	High active
Argentina	1189	27.1 (24–31)	35.4 (32–39)	37.5 (34–41)	26.3 (23–30)	34.9 (31–39)	38.6 (35–42)
Australia	2642	14.3 (12–16)	20.0 (18–22)	65.8 (63–68)	20.1 (18–22)	28.5 (26–31)	51.4 (49–54)
Belgium	1922	37.3 (34–40)	25.4 (23–28)	37.2 (34–40)	48.7 (46–52)	29.4 (27–32)	21.9 (19–25)
Brazil	981	25.6 (22–29)	34.9 (31–39)	39.5(35–44)	34.3 (30–38)	52.7 (48–57)	13.0(10–16)

Canada	2626	12.3 (10–14)	23.2 (21–26)	64.4 (62–67)	15.1 (13–17)	30.1 (28–33)	54.8 (52–58)
China	1593	7.4 (6–9)	33.4 (30–37)	59.1 (56–63)	6.4 (5–8)	37.4 (34–41)	56.2 (53–60)
Colombia	2974	16.5 (15–18)	23.7 (22–26)	59.7 (57–62)	23.1 (21–25)	31.2 (29–34)	45.7 (43–48)
Czech Rep	7468	9.8 (9–11)	22.1 (21–23)	68.2 (67–70)	10.0 (9–11)	32.2 (31–34)	57.7 (56–59)

Hong Kong	4657	13.8 (12–15)	46.5 (44–48)	39.8 (38–42)	16.8 (15–18)	54.6 (53–57)	28.6 (27–30)
India	1004	22.5 (19–26)	37.8 (34–42)	39.8 (36–44)	24.3 (21–28)	39.6 (35–44)	36.1 (32–40)
Japan	4618	41.1 (39–43)	33.1 (31–35)	25.8 (24–28)	45.6 (44–48)	37.8 (36–40)	16.7 (15–18)
Lithuania	2210	15.9 (14–18)	28.3 (26–31)	55.7 (53–59)	14.0 (12–16)	37.5 (35–40)	48.4 (45–51)

New Zealand	1449	8.0 (6–10)	18.0 (15–21)	74.0 (71–77)	16.5 (14–19)	31.3 (28–35)	52.2 (49–56)
Norway	1625	25.1 (22–28)	29.2 (26–32)	45.8 (42–49)	27.2 (24–30)	38.1 (35–41)	34.7 (31–38)
Portugal	1435	28.9 (26–32)	27.0 (24–30)	44.1 (40–48)	23.4 (20–27)	30.1 (27–33)	46.4 (43–50)
Saudi Arabia	974	42.8 (38–47)	36.8 (33–41)	20.2 (17–24)	37.3 (33–42)	30.7 (27–35)	32.2 (28–36)

Spain	1541	24.3 (21–27)	29.8 (27–33)	45.9 (42–49)	24.1 (21–27)	42.7 (39–46)	33.3 (30–37)
Sweden	1254	25.6 (22–29)	31.8 (28–35)	42.5 (39–46)	22.2 (19–25)	42.7 (39–47)	34.9 (31–39)
Taiwan	4773	41.3 (39–43)	28.2 (26–30)	30.5 (29–32)	43.3 (41–45)	37.6 (36–40)	19.1 (18–21)
USA	4587	13.6 (12–15)	19.2 (18–21)	67.2 (65–69)	18.2 (17–20)	25.0 (23–27)	56.7 (55–59)

* Age and gender standardised to the world population 2002 [US Census Bureau 2002 [16]; Doll 1966, [17].

† Australia: Age 18–64 only; Hong Kong: Age 20–64 only; Japan: Age 18–39 only; Portugal: Age 40–65 only

## note that sample sizes differ from Table 1 due to minimal missing data on some physical activity questions

Further analyses compared these data by age group, assessing younger (18–39 years) and older adults (40–65 years). Physical activity declined with age (at least a difference of 10% in "high" PA prevalence in the older age group compared to the younger age group) in 11 out of 19 estimates for males, but only three countries showed this difference among females (Table 4). Although there was a general decline across age groups, for some countries, rates of physical activity remained high in the older age group (for example, New Zealand males and females), or even increased, compared to the younger age group (Chinese and Hong Kong, SAR females).

**Table 4:** Prevalence* of International Physical Activity Questionnaire categories by age group by gender and country^†^; International Prevalence Study, 2002–2004.

Country	% Prevalence											
	
	Male						Female					
		
	18–39			40–65			18–39			40–65		
				
	Low	Mod	High #	Low	Mod	High	Low	Mod	High	Low	Mod	High
			
Argentina	26	32	41	28	40	33	21	34	45	34	36	30
Australia	13	17	70	16	23	61	19	28	53	21	30	49
Belgium	33	27	41	43	24	33	44	33	23	54	25	21
Brazil	17	36	46	36	33	31	31	54	15	38	51	11
Canada	10	22	69	15	25	59	13	30	57	18	31	51
China	7	30	63	8	37	55	8	40	53	5	35	60
Colombia	14	17	69	20	32	48	22	30	49	25	33	42
Czech Rep	7	20	73	13	25	62	9	31	60	12	34	55

Hong Kong	14	45	42	14	48	38	19	57	25	15	52	33
India	25	22	53	19	57	23	27	28	45	21	54	25
Japan	41	33	26	-	-	-	46	38	17	-	-	-
Lithuania	12	24	63	21	33	46	13	36	51	15	40	45

New Zealand	7	17	76	9	19	71	16	32	53	18	31	52
Norway	21	28	51	30	30	40	27	36	37	28	41	31
Portugal	-	-	-	29	27	44	-	-	-	23	30	46
Saudi Arabia	38	35	27	49	39	12	35	32	32	39	29	32

Spain	17	31	52	34	28	38	24	41	36	24	45	30
Sweden	23	30	47	29	34	37	20	40	40	25	46	29
Taiwan	42	27	31	41	30	29	45	36	19	41	39	20
USA	11	16	72	16	23	61	18	26	56	19	24	57

# low, moderate [mod] and high active IPAQ categories

* Age-and gender – standardized to the world population 2002 [US Census Bureau 2002 [16]; Doll 1966, [17].

† Australia: Age 18–64 only; Hong Kong: Age 20–64 only; Japan: Age 18–39 only; Portugal: Age 40–65 only

**Figure 1:**
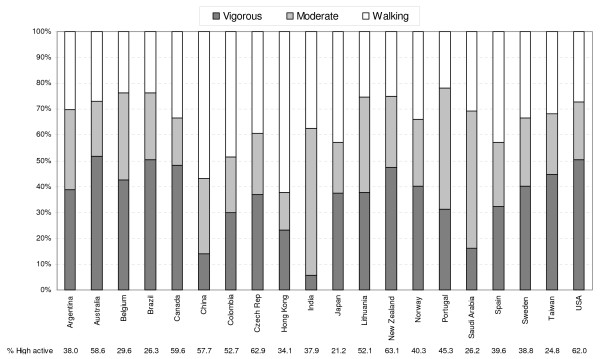
**Prevalence of high activity, and proportions of total physical activity for each country derived from walking, moderate- and vigorous-intensity activity**.

Figure 1 shows the relative contributions of walking, moderate-intensity, and vigorous-intensity physical activity to total MET-minutes/week for each country. In all countries at least 20% of total MET-minutes/week was accrued through walking, and walking comprised at least 50% of total MET-minutes/week in Hong Kong SAR, and China. By contrast, the contribution of vigorous-intensity activity to total MET-minutes/week varied widely among countries. For example, vigorous-intensity activity contributed less than 5% of total MET-minutes/week in India, but more than 45% of total MET-minutes/week in Australia, Brazil, Canada, New Zealand and the USA.

## Discussion

International comparisons of key non-communicable disease (NCD) risk factors, such as obesity and tobacco use, are commonplace [19,20], but comparisons of physical activity prevalence among countries have not been possible due to the lack of standardised and validated instruments. Previous reviews have demonstrated very different prevalence estimates across countries, and these differences were as likely to be due to variations in questions and survey methods as they were to true differences in prevalence [21,22]. The present study reports population-level prevalence estimates and patterns of physical activity in a diverse set of countries, using a comparable, reliable and validated survey instrument. In this study, the IPAQ short form was administered to over 52,000 adults aged 18–65 years, using a standard protocol in 20 countries.

The results show substantial variation in the population estimates of meeting the IPAQ "high active" category, a threshold developed to reflect an amount greater than than standard recommended levels, but more suited for use with a multiple domain measure such as IPAQ. Eight of the 20 countries had "high activity" rates over 50%, and these countries came from several continents. We also observed different patterns by gender and age, with most countries showing younger men more active than younger women, but this gender difference was less marked among older adults. Further, in these countries, age-related declines in physical activity were much more frequently observed among men than among women.

As shown in Figure 1, countries achieved high physical activity in different ways, with four of the most active countries (Australia, Canada, New Zealand, USA) showing a greater volume of vigorous-intensity physical activity relative to moderate-intensity activity and walking. These same countries seem to have relatively well developed facilities for recreational activity and a history of long-term promotion of exercise. Four of the countries with substantial rates of high physical activity had more than 30% of overall physical activity derived from walking (Canada, China, Colombia, Czech Republic) suggesting that countries with an infrastructure or culture that supports walking can achieve high levels of physical activity with lesser contribution from vigorous activity. However, substantial proportions from walking (Hong Kong SAR, Japan, Spain, Taiwan) and vigorous activity (Belgium, Brazil, Taiwan) were also found in countries with low overall physical activity prevalence rates (< 30% in the 'high' category), so there is no indication that an emphasis on promoting one domain of activity will lead to high levels of overall physical activity at the population level. One conclusion from these results is that different patterns of physical activity are associated with high prevalence estimates, so countries could tailor physical activity promotion strategies to local infrastructure, available programs, and culture.

There are a few European multi-country physical activity surveys that provide a context for interpreting present results. The 2002 Eurobarometer study used the IPAQ instrument [13] and identified low prevalence estimates for Belgium and Sweden, similarly to IPS and reported the highest levels of physical activity in the Netherlands and Germany (these two countries did not participate in IPS). However, earlier European-only studies had observed slightly different rankings [14,23]. A Pan-European Union Survey on Consumer Attitudes to Physical Activity had found a similar low prevalence of activity in Belgium and Portugal, but reported a high prevalence rate in Sweden [23]. Another study used information from the 2002–3 WHO World Health Survey to collect IPAQ short-form physical activity data from 51 countries, mostly from population samples in developing countries [24]. Physical inactivity prevalence data from the four comparable countries were remarkably similar to the findings in this IPS study; for example, inactivity rates were low for China and the Czech Republic [around 10% in both the IPS and the World Health Survey], and high levels of inactivity reported in both surveys for Brazil [close to 30% inactive in both studies], and close to 25% for Spanish adults in both studies. The similarity of these estimates was noteworthy, despite different survey methods, suggesting that IPAQ data may be consistent, at least within country.

One interesting further comparison can be made from representative cross sectional data of adolescents in Europe and North America through the contemporaneous 2001–2002 Health Behaviour in School-aged children survey (HBSC) [25]. Looking at the group closest to the IPS age group (the 15 year old samples) the countries with the most active boys and girls included USA, Canada, Czech Republic and Lithuania in the upper quartile, and Belgium, Norway and Portugal in the lowest quartile. This distribution for physical activity among adolescents was similar to that observed in the IPS among adults from the same countries.

The findings from this study indicate that the majority of the population in most participating countries or regions appeared to undertake at least a moderate amount of physical activity when assessed using the multi-domain IPAQ. This suggests that most adults in these countries are obtaining some activity, yet the global problem of rising prevalence of obesity remains. Thus, it appears total physical activity in most countries remains insufficient to ensure energy balance and prevent obesity [26] or that the ratio of energy expenditure to dietary intake is unbalanced to maintain weight stability.

Strengths of IPAQ include its measurement of multiple domains, and the separate assessment of walking behaviour, compared to many current PA surveillance systems [27]. There are some limitations with IPAQ, including difficulties with respondents in distinguishing moderate and vigorous activities. It is also well recognised that self-reported measures can over-estimate physical activity [28], and the IPAQ may do this more than other surveys [29-31]. Although there is a benefit in IPAQ assessing multiple domains of physical activity as part of global surveillance, it could contribute to higher overall PA estimates than previous surveys that captured leisure-time activity alone [15]. There is also the possibility of differential measurement error using IPAQ, with some countries or population subgroups potentially giving relatively accurate estimates of their behaviour, while other populations may over-estimate or under-estimate their physical activity; this between-country variability appeared even greater in the World Health Survey, which was comprised of mostly developing countries [24]. The samples here were large-scale population samples, but eight of them were regional, not national samples, so these prevalence estimates cannot be generalised to the country level (for example, to all of China or Argentina). Based on education level (Table 1), these samples showed similar or slightly higher educational attainment to national or regional levels for those countries, suggesting comparability on this attribute.

Other methodological issues in the IPS included the variations in response rates across countries, and despite many efforts to standardize protocols, there were undoubtedly important differences in implementation. While all surveys were reviewed for consistency in the translation and cultural adaptation for participating countries, variations in how respondents understood the survey may have contributed to difficulties in interpreting findings of this international prevalence study. Though it is likely impossible to eliminate all challenges to the comparability of survey results across countries, subsequent surveillance studies could benefit from additional training of investigators, development of more rigorous translation and adaptation procedures, on-site inspections for quality control of data collection, centralized data entry system, and other methods for enhancing data quality. Furthermore, in low literacy populations, standardised showcards or illustrations should be used to depict types or intensity of different physical activities, but these cannot be used with telephone-based survey administration.

In addition to the abovementioned methodological issues, concerns remain with self report measures. Whilst they remain the most feasible and affordable instruments for global surveillance, objective population measures of physical activity, such as pedometers [32] or accelerometers [33,34], may be beneficial to determine if differences across countries and between groups revealed in the present study represent true differences in physical activity behaviour.

The International Prevalence Study on Physical Activity provided data that allow 20 countries to be compared on physical activity behaviours for the first time. For some countries, these data represent their first large scale measurement of physical activity. Several countries have adopted the IPAQ and IPS methods as their national or regional surveillance system, and these data contribute to current WHO and European surveillance systems. A major contribution of IPS was to produce internationally comparable physical activity prevalence estimates and to demonstrate the feasibility of standardised data collection on physical activity using a common protocol. Though questions remain about the precision of the derived prevalence estimates, an important next step is the continued use of established instruments for the collection and monitoring of physical activity as part of ongoing non-communicable disease risk factor surveillance systems. Ongoing research will compare the IPAQ results with the Global Physical Activity Questionnaire (GPAQ), the World Health Organization's instrument also being used for international surveillance [21,35], and compare both of these against objective measures.

The results from this international surveillance study on physical activity can provide useful baseline data, at least within countries, and studies could be repeated to ascertain population trends in physical activity. One important feature of a useful health monitoring system is the use of consistent measures over time [36]. Country-specific data and trends can be used to monitor efforts to promote physical activity and improve public health. Although surveillance data alone are not sufficient to motivate or guide the implementation of national policy, consistent trend data are an essential underpinning of the case for public health action.

## Competing interests

The authors declare that they have no competing interests.

## Authors' contributions

AB supervised the data collection and management, conceptualized the paper, and carried out primary writing and interpretation of the paper, and is guarantor for it. FB coordinated the recruitment of countries and the IPS group, contributed to the conceptualization of the study, primary drafting and interpretation of the paper. CLC and BEA were responsible for country inclusion and study protocols, IPAQ translation and contributed to the conceptualization of the study, writing and interpretation of the paper. JFS, MH, MS, MP contributed to the conceptualization of the study, assisted with the writing and interpretation of the paper. TC and HRB analyzed the data, and assisted with interpretation and writing. IPS centers coordinated study implementation, IPAQ translation and data collection in each country, following standard protocols.

## Appendix 1

### IPS Collaborating research groups in each country

#### Country level authors

Argentina: Díaz Colodrero G, Bazan N, Kunic H; Asociación Metropolitana de Medicina del Deporte, Buenos Aires

Australia: Bauman A, Merom D, Smith B. Centre for Physical Activity and Health, University of Sydney

Belgium: De Bourdeaudhuij I, Lefevre J., Philippaerts R Department of Movement and Sport Sciences, Ghent University and Faculty of Kinesiology and Rehabilitation Sciences, K.U. Leuven

Brazil: Matsudo SM, Matsudo VR, CELAFISCS – Physical Fitness Research Center (Celfasics), São Caetano do Sul, São Paulo.

Canada: Craig CL, Cameron C, Canadian Fitness and Lifestyle Research Institute, Ottawa

China PRC: Yang Li, Hua Fu, Department of Preventive Medicine, School of Public Health, Fudan University, Shanghai

Colombia Gómez LF, Health Division, Fundacion Fes Social, Bogota.

Czech Republic: Fromel K, Mitas J. Centre for Kinanthropology Research, Palacky University

Hong Kong SAR China: Macfarlane D, Bacon-Shone J, The University of Hong Kong, Pokfulam.

India: Reddy SK, Joshi P, Goenka S, Prabhakaran D, All India Institute of Medical Sciences, New Delhi

Japan: Katsumura T, Murase N. Department of Sports Medicine for Health Promotion, Tokyo Medical University

Lithuania: Volbekiene V, Baubliene R. Lithuanian Academy of Physical Education (LAPE)

New Zealand: McLean G, Sport and Recreation New Zealand (SPARC), Carr H, Ministry of Health, Wellington

Norway: Tomten H, Directorate for Health and Social Affairs; Anderssen SA, Norwegian School of Sport Sciences, Oslo

Portugal: Sardinha L, Technical University of Lisbon. Mota J, Faculty of Sports-University of Porto

Saudi Arabia: Al-Hazzaa HM Exercise Physiology Laboratory, King Saud University, Riyadh

Spain: Serra Majem L, Roman B, Community Nutrition Research Center, Barcelona Science Park, University of Barcelona;

Sweden: Sjöström M, Hagströmer M, Bergman P. Department of Biosciences and Nutrition, Karolinska Institutet, Sweden

Taiwan: Yiing Mei Liou, National Yang-Ming University; Yung-Tai Hung, National Taiwan University.

USA: Ainsworth BE, Department of Exercise and Wellness, Arizona State University; Hipp D, Physical Activity and Health Branch, U.S. Centers for Disease Control and Prevention Atlanta, Georgia USA

## Appendix 2 – Criteria for inclusion in the IPS project

### Sample Population and sample size

A representative population sample of at least 1500 adults aged 18 to 65 years was suggested. This sample should be representative of national populations or of a sizeable portion or region(s) within a country (defined as a population of over one million). In some circumstances samples which did not meet either of these criteria but that which the country itself would regard as representing the national population were considered acceptable. Samples were to be selected using simple random sampling and simple random sampling proportional to size.

### Data Collection timeframes

Data collection was planned for Spring or Fall (Autumn) seasons in 2002/03 (if data were collected across 12 months only these months would be used) as these seasons were deemed most comparable across countries and less likely to be affected by extreme weather. In special cases, other months were accepted because meteorological data were examined and weather conditions were considered comparable.

### Response Rates and Sample Characteristics

To assess representativeness, response rates were required, as well as the most recent Census data providing information on age, sex, education and ethnicity for the sample population (country or region).

### Questionnaire Administration

Telephone interview, face-to-face interview, or self-administration were permitted. No proxy interviews were accepted.

### Obtaining Informed Consent

Informed consent (using local established procedures) must be obtained.

### Questionnaire

IPAQ short form was mandatory and must be used with core demographic questions. Details of the context of data collection (e.g., embedded in a health survey or a stand alone physical activity survey) were required as well as question order and details of any other additional questions on physical activity. No changes to the short IPAQ form other than cultural adaptations were permitted. Any other physical activity questions were to occur after the IPAQ short form was completed (otherwise the sample should be randomly split in half with one half the sample receiving the IPAQ questions first and the second half receiving any other physical activity questions first). Core demographic information comprised: age, gender, education, current employment status, and city size.

### Translation and Cultural Adaptation

Guidelines for cultural adaptation and translation were provided and are available at . A copy of the final survey and English back-translation was required.
